# Functional Genomic Analysis of a *RUNX3* Polymorphism Associated With Ankylosing Spondylitis

**DOI:** 10.1002/art.41628

**Published:** 2021-05-02

**Authors:** Matteo Vecellio, Liye Chen, Carla J. Cohen, Adrian Cortes, Yan Li, Sarah Bonham, Carlo Selmi, Matthew A. Brown, Roman Fischer, Julian C. Knight, B. Paul Wordsworth

**Affiliations:** ^1^ NIHR Oxford Musculoskeletal Biomedical Research Unit Botnar Research Centre Nuffield Orthopaedic Centre NIHR Oxford Comprehensive Biomedical Research Centre University of Oxford Oxford UK; ^2^ John Radcliffe Hospital Wellcome Centre for Human Genetics University of Oxford Oxford UK; ^3^ The First Affiliated Hospital of Xiamen University and Xiamen University School of Medicine Xiamen China; ^4^ Target Discovery Institute University of Oxford Oxford UK; ^5^ IRCCS Humanitas Research Hospital Milan Italy; ^6^ NIHR Guy’s and St. Thomas’ Biomedical Research Centre, London, UK, and University of Queensland Brisbane Queensland Australia; ^7^ Wellcome Centre for Human Genetics University of Oxford Oxford UK

## Abstract

**Objective:**

To investigate the functional consequences of the single‐nucleotide polymorphism *rs4648889* in a putative enhancer upstream of the *RUNX3* promoter associated with susceptibility to ankylosing spondylitis (AS).

**Methods:**

Using nuclear extracts from Jurkat cells and primary human CD8+ T cells, the effects of *rs4648889* on allele‐specific transcription factor (TF) binding were investigated by DNA pull‐down assay and quantitative mass spectrometry (qMS), with validation by electrophoretic mobility shift assay (EMSA), Western blotting of the pulled‐down eluates, and chromatin immunoprecipitation (ChIP)–quantitative polymerase chain reaction (qPCR) analysis. Further functional effects were tested by small interfering RNA knockdown of the gene for interferon regulatory factor 5 (IRF5), followed by reverse transcription–qPCR (RT‐qPCR) and enzyme‐linked immunosorbent assay (ELISA) to measure the levels of IFNγ messenger RNA (mRNA) and protein, respectively.

**Results:**

In nuclear extracts from CD8+ T cells, results of qMS showed that relative TF binding to the AS‐risk A allele of *rs4648889* was increased 3.7‐fold (*P* < 0.03) for Ikaros family zinc‐finger protein 3 (IKZF3; Aiolos) and components of the NuRD complex, including chromodomain helicase DNA binding protein 4 (CHD4) (3.6‐fold increase; *P* < 0.05) and retinoblastoma binding protein 4 (RBBP4) (4.1‐fold increase; *P* < 0.03). In contrast, IRF5 bound significantly more to the AS‐protective G allele compared to the AS‐risk A allele (fold change 8.2; *P* = 0.003). Validation with Western blotting, EMSA, and ChIP‐qPCR confirmed the differential allelic binding of IKZF3, CHD4, RBBP4, and IRF5. Silencing of *IRF5* in CD8+ T cells increased the levels of IFNγ mRNA as measured by RT‐qPCR (*P* = 0.03) and IFNγ protein as measured by ELISA (*P* = 0.02).

**Conclusion:**

These findings suggest that the association of *rs4648889* with AS reflects allele‐specific binding of this enhancer‐like region to certain TFs, including IRF5, IKZF3, and members of the NuRD complex. IRF5 may have crucial influences on the functions of CD8+ lymphocytes, a finding that could reveal new therapeutic targets for the management of AS.

## INTRODUCTION

Ankylosing spondylitis (AS) is a form of spondyloarthropathy that is characterized by prominent axial skeletal enthesitis, spinal fusion, and deformity. It is highly heritable, but its genetic etiology is complex; even its strong association with the major histocompatibility complex (MHC) reflects its association not just with *HLA–B27* but also with numerous additional MHC class I and class II immune‐response genes (1). Outside the MHC, more than 100 genetic influences have been identified in genome‐wide association studies (GWAS) (2, 3), but only a few of the associated single‐nucleotide polymorphisms (SNPs) actually produce amino acid substitutions with functional effects. For example, *rs11209026* in *IL23R* results in impaired signaling through the interleukin‐23 (IL‐23) receptor, which is protective against AS (4). Moreover, *rs30187* in *ERAP1* alters the trimming of peptide antigens by endoplasmic reticulum aminopeptidase 1 (ERAP‐1), which functions synergistically with *HLA–B27* in the MHC class I antigen presentation pathway to influence susceptibility to AS (5, 6). In contrast, most disease‐associated SNPs are believed to operate through their effects on gene expression, which involves cell type–specific epigenetic mechanisms such as the differential binding of transcription factors (TFs) or microRNAs (7).

We and others have previously reported strong associations between AS and a cluster of SNPs upstream of the *RUNX3* gene (encoding RUNX family transcription factor 3) close to a putative regulatory element with “enhancer‐like” characteristics (5, 8). We have shown that the AS‐protective *rs4648889* G allele was associated with higher *RUNX3* expression in CD8+ T cells than the disease‐associated A allele. In vitro, the binding of TFs from nuclear extracts was influenced by *rs4648889*, and one member of the interferon regulatory factor (IRF) family of TFs, IRF4, appeared to be involved (8). IRF5, which is another closely related member of this family, shares similar DNA binding characteristics with IRF4, and has also been previously implicated in several autoimmune/inflammatory diseases (9, 10). IRF5 plays a key role in macrophage function and its polarization toward the M1 (inflammatory) phenotype (11), but the potential activity of IRF5 in T cells is less well described. RUNX3 is itself also a TF, and plays a key regulatory role in several lineage‐specific developmental pathways, including T cells. It is involved in the pathophysiology of infections, immunity, and cancer (12, 13).

As the regulatory effects of TFs are frequently mediated through complexes containing multiple components, rather than a single TF, we decided to investigate the effects of *rs4648889* on protein–DNA complex formation using a hypothesis‐free approach. We used DNA pull‐down assays combined with quantitative mass spectrometry (qMS) to define the full range of interacting TF partners binding at *rs4648889* (8, 14). Our results reveal the involvement of IRF5, and demonstrate that silencing *IRF5* in CD8+ T cells may have important functional consequences. We also demonstrate significant differential binding at *rs4648889* for IKZF3 (the Ikaros family zinc‐finger protein also known as Aiolos), which plays a major role in lymphocyte differentiation and function (15), and also binding of several factors of the NuRD complex, which is often physically associated with Aiolos and involved in chromatin remodeling (16).

## MATERIALS AND METHODS

#### Cell culture, isolation of CD8+ T cells, and preparation of nuclear extracts

Jurkat cells were cultured in RPMI medium supplemented with 10% fetal bovine serum, penicillin/streptomycin, and L‐glutamine. CD8+ T cells were isolated from human peripheral blood mononuclear cells obtained from buffy coat, using a CD8+ T cell isolation kit (catalog no. 130‐096‐495; Miltenyi, UK); subjects provided informed consent for use of these blood samples (obtained from NHS Blood and Transplant, Oxford University Hospitals NHS Foundation Trust). CD8+ T cells were resuspended at 1 × 10^6^/ml in prewarmed RPMI medium supplemented with 10% fetal bovine serum, penicillin/streptomycin, and L‐glutamine. The cells were harvested after 4 hours in resting conditions. Nuclear extract was prepared using an NE‐PER Nuclear reagent and cytoplasmatic extraction reagents (catalog no. 78833; ThermoFisher Scientific), in accordance with the manufacturer’s instructions.

#### DNA‐affinity capture

**Figure 1:**
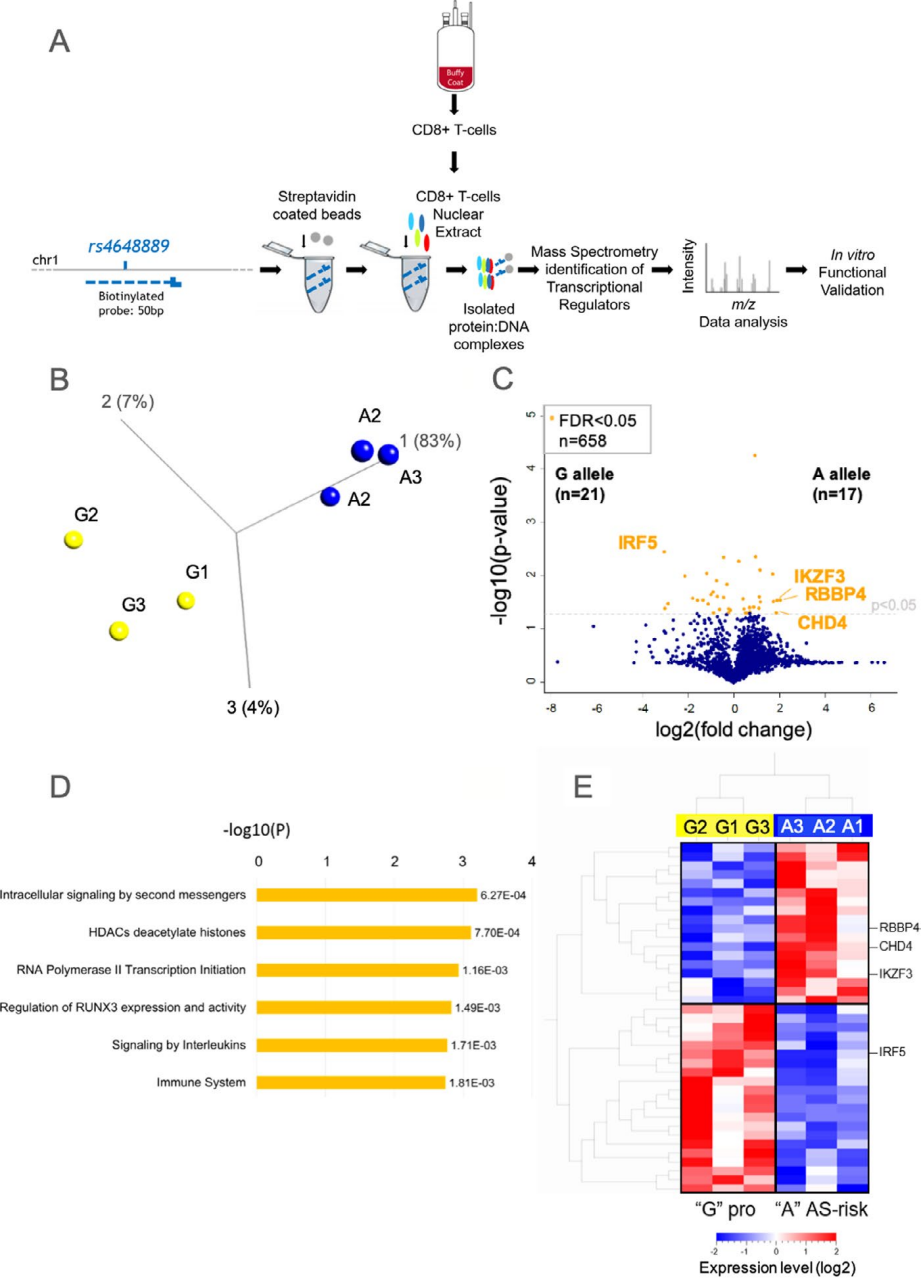
Identification of proteins bound to the *rs4648889* locus in nuclear extracts from CD8+ T cells, using DNA‐affinity capture assay/quantitative mass spectrometry (qMS). **A**, Workflow diagram of the experimental approach. **B**, Principal components analysis of variance in protein levels in 3 replicated experiments, assessing binding to the ankylosing spondylitis (AS)–protective G allele versus the AS‐risk A allele. **C**, Volcano plot showing the complete set of 658 proteins identified as showing differential binding in 3 different qMS experiments. Orange dots represent the proteins showing statistically significant variance at a false discovery rate (FDR) of <0.05. **D**, Reactome pathway analysis of the functional pathways associated with statistically significant proteins. **E**, Unsupervised hierarchical clustering of the statistically significant proteins (FDR <0.05) showing differential binding between the AS‐protective and AS‐risk alleles. RBBP4 = retinoblastoma binding protein 4; CHD4 = chromodomain helicase DNA binding protein 4; IKZF3 = Ikaros family zinc‐fingerprotein 3; IRF5 = interferon regulatory factor 5.

An overview of the experimental approach used for DNA‐affinity capture assay is shown in Figure 1 The 50‐bp sense and antisense oligonucleotides centered around the A or G allele of *rs4648889* were obtained from Eurofins Genomics (sequences of the oligonucleotides are listed in Supplementary Table 1, available on the *Arthritis & Rheumatology* website at http://onlin​elibr​ary.wiley.com/doi/10.1002/art.41628/​abstract). In these experiments, 100 n*M* antisense single‐stranded oligonucleotides (50 bp) were 3′‐end biotinylated, mixed, and annealed at room temperature (RT) for 1 hour with the sense oligonucleotide. Streptavidin‐coated magnetic beads (Dynabeads M‐280, catalog no. 11205D; ThermoFisher Scientific) were equilibrated by 6 washes with wash buffer (10.0 m*M* Tris HCl, pH 7.4, 2.0*M* NaCl, 1 m*M* EDTA). Biotin‐labeled DNA was incubated with streptavidin‐coated beads for 1 hour at RT, on a rotary wheel. Three successive washes were performed to eliminate the unbound biotinylated DNA. Nuclear extract (500 μg) from CD8+ T cells was preincubated on ice for 20 minutes in electrophoretic mobility shift assay (EMSA) binding buffer (100 m*M* Tris, 500 m*M* KCl, 10 m*M* dithiothreitol [pH 7.5]) and then incubated with beads for 1 hour at 21°C on a rotary wheel. The beads were then stringently washed 6 times: once with 500 μl of EMSA binding buffer, 3 times with wash buffer plus 0.1% Tween 20, and twice with 50 m*M* NH_4_HCO_3_. Beads were then resuspended in sample buffer containing 10 m*M* EDTA, 1% sodium dodecyl sulfate (SDS), and benzonase (1 unit) for 60 minutes at RT on a rotary wheel. Magnetic separation was used to separate the DNA–protein complexes from the beads.

#### Mass spectrometry analyses

Protein samples were prepared for liquid chromatography tandem mass spectrometry (LC‐MS/MS) analysis using tryptic digestion, as described previously (17). Briefly, proteins were reduced and alkylated (with 1‐4 dithiothreitol/iodoacetamide) before digestion with trypsin (Promega) and desalting of peptides using C18 material (Sola; ThermoFisher). Thereafter, the peptides were analyzed on a nano LC‐MS/MS platform consisting of a Q‐Exactive mass spectrometer and nano ultra‐performance LC mass spectrometer (both from ThermoFisher) (18). Chromatographic separation of peptides was achieved on Easyspray columns (75 μm × 500 mm) using a gradient spanning from 5% DMSO in 0.1% formic acid in 5% acetonitrile to 5% DMSO in 0.1% formic acid in 35% acetonotrile. The MS parameters used have been described previously (18).

Quantitative data were derived from the number of MS/MS spectra per peptide (spectral counting) or the integrated peak area of the ion chromatogram of a specific peptide as reported by ProgenesisQI (Waters, version 2.0) using default parameters. Identification of proteins was generated with the use of the Mascot search engine based on a false discovery rate (FDR) of 1% and peptide score cutoff of 20, checked against the UniProt human protein database. All proteomics data are publicly available through the Proteomics Identification Database (PRIDE consortium; https://www.ebi.ac.uk/pride/​archi​ve/) (19).

#### Visualization and analysis of proteomics data

Proteomics data were visualized using a Qlucore Omics Explorer (version 3.7) in order to carry out principal components analysis and unsupervised hierarchical clustering. The Reactome database (20) was used to investigate the functional pathways (i.e., Gene Ontology [GO] categories). The R package was used to create volcano plots. The Search Tool for the Retrieval of Interacting Genes/Proteins (STRING; version 11.0), a database of known and predicted protein–protein interactions, was used to define the protein–protein interactions among the 38 differentially abundant proteins identified by DNA pull‐down assay/qMS analysis.

#### Epigenetic database interrogation

Epigenetic data from Roadmap Epigenomics Projects (http://epige​nomeg​ateway.wustl.edu) (21) was used to analyze the region encompassing SNP *rs4648889*, in particular the chromatin immunoprecipitation–sequencing (ChIP‐seq) peaks for the TFs IKZF and chromodomain helicase DNA binding protein 4 (CHD4) on lymphoblastoid cell lines.

#### EMSA

EMSA were performed as previously described (8). Briefly, the DNA probes were mixed and annealed at RT for 1 hour. For supershift assays, 5 μg of nuclear extract obtained from Jurkat cells was first incubated (for 20 minutes) with a specific antibody, and then the nuclear extract–antibody complex was incubated (for 20 minutes) with biotinylated DNA and run on retardation gels. The full list of antibodies and DNA probes is provided in Supplementary Tables 1 and 2 (available on the *Arthritis & Rheumatology* website at http://onlin​elibr​ary.wiley.com/doi/10.1002/art.41628/​abstract).

#### Western blot assay

Eluted samples from the qMS experiments were separated by SDS–polyacrylamide gel electrophoresis, transferred to nitrocellulose membranes (Bio‐Rad), and incubated overnight at 4°C with various primary antibodies against IKZF3, CHD4, retinoblastoma binding protein 4 (RBBP4), and IRF5 (see Supplementary Table 2 [http://onlin​elibr​ary.wiley.com/doi/10.1002/art.41628/​abstract]). An appropriate horseradish peroxidase–conjugated secondary antibody was used, and signals were detected using the enhanced chemiluminescence method (ThermoFisher Scientific). Image quantitation was performed using ImageJ.

#### ChIP–quantitative polymerase chain reaction (qPCR) analysis

Chromatin was sonicated with Bioruptor Pico (Diagenode) and fragment sizes were analyzed on a 2% agarose gel. ChIP samples were prepared using the iDeal ChIP‐seq kit for Transcription Factors (catalog no. C01010055; Diagenode). For each ChIP sample, 2.5 × 10^6^ CD8+ T cells were used. Three independent qPCR experiments were performed using allele‐specific primers for *rs4648889* (see ref. 8 for specific primer sequences). We used CD8+ T cells (n = 3 samples) of known genotype (heterozygous for *rs4648889*) from buffy coat blood cones to compare the impact of the AS‐risk and AS‐protective alleles on relative enrichment. We normalized all of our ChIP‐qPCR data against a 1% input control, in accordance with the manufacturer’s instructions.

Data were visualized with Prism version 8.0.2. The following ChIP‐grade antibodies were used: anti‐RBBP4 (ab79416; Abcam), anti‐CHD4 (14173‐1‐AP; Proteintech Europe), anti‐IKZF3 (ab139408; Abcam), anti‐IRF5 (E1N9G, rabbit monoclonal antibody 13496; Cell Signaling Technology), and an IgG antibody (K02041008; Diagenode).

#### *IRF5* silencing

Primary human CD8+ T cells were transfected with small interfering RNA (siRNA) targeting *IRF5* or with a scrambled control siRNA (both from Dharmacon), using the Neon transfection system (ThermoFisher Scientific). The cells were then stimulated with anti‐CD2/anti‐CD3/anti‐CD28 beads (Miltenyi, UK) and a proinflammatory cytokine cocktail (consisting of interleukin‐2 [IL‐2], IL‐1β, IL‐6, and IL‐23; all from PeproTech). Three days after transfection, the supernatant was collected for enzyme‐linked immunosorbent assay (ELISA) analysis of interferon‐γ (IFNγ), and cells were lysed in TRIzol for RNA isolation and qPCR analysis of *RUNX3*, *IRF5*, and IFNγ messenger RNA (mRNA) expression, using a TaqMan gene expression assay.

#### Statistical analysis

Statistical analysis for the volcano plot and hierarchical clustering data was performed using base R. Student’s 2‐tailed *t*‐test was used to determine statistically significant differences between groups, calculated using GraphPad Prism software (version 8.01).

## RESULTS

#### SNP‐based capture of TFs and identification of differentially bound proteins by label‐free MS

We hypothesized that the effects of *rs4648889* on disease association and differential gene expression can be attributed to allele‐specific TF binding in CD8+ T cells. We first sought to identify which TFs have the capacity to bind in an allele‐specific manner, using a highly sensitive DNA pull‐down approach with analysis using label‐free MS. Nuclear proteins from freshly isolated CD8+ T cells were incubated with *rs4648889*‐centered DNA oligonucleotide baits corresponding to the 2 naturally occurring alleles, followed by qMS analysis in 3 independent experiments (Figure 1A).

Principal components analysis demonstrated that there were clear clusters of proteins preferentially binding the different *rs4648889* alleles (Figure 1B). There was significant differential binding for 38 proteins (FDR <0.05) between the AS‐risk A allele and the AS‐protective G allele, as shown in the volcano plot (Figure 1C and Supplementary Table 3, available on the *Arthritis & Rheumatology* website at http://onlin​elibr​ary.wiley.com/doi/10.1002/art.41628/​abstract). Reactome pathway analysis revealed significant enrichment for proteins involved in immunity, chromatin remodeling/histone deacetylation, RNA polymerase II transcription initiation, and regulation of *RUNX3* expression and activity (Figure 1D). Unsupervised hierarchical clustering analysis demonstrated distinct DNA–protein “interactome” profiles for the 2 *rs4648889* alleles (Figure 1E).

#### Identification of IKZF3 and NuRD cofactors by qMS analysis

**Figure 2:**
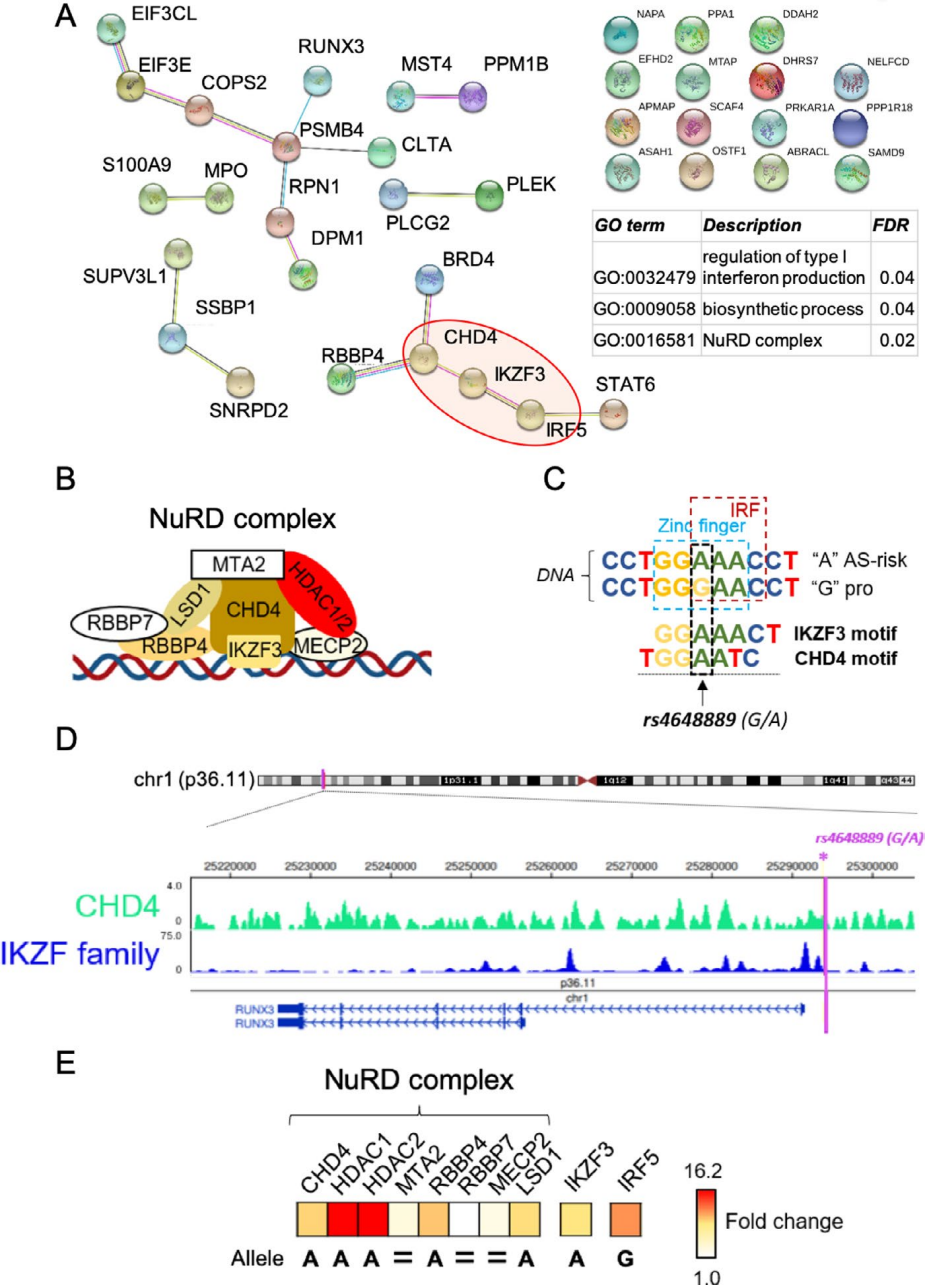
Identification of IKZF3, CHD4, and NuRD complex factors in binding the genomic region spanning *rs4648889*. **A**, Protein–protein interaction analysis of the 38 factors identified as significant by DNA pull‐down/qMS and related Gene Ontology (GO) analysis. **B**, Illustration showing the binding of IKZF3 (Aiolos) and NuRD complex factors to DNA. **C**, DNA binding motifs of IKZF3 and CHD4 (verified in the ENCODE Factorbook [21]) that overlap the *rs4648889*‐encompassing locus. **D**, Chromatin immunoprecipitation–sequencing peak signals for CHD4 and IKZF proteins on GM12878 cell lines, as revealed by interrogation of the Epigenome database. Purple vertical line indicates the location of the *rs4648889* genetic variant. **E**, Label‐free quantitation of IKZF3, NuRD complex factors, and IRF5 identified by qMS. The heatmap represents the differential binding of each protein, quantified as fold change in expression. pro = protective; HDAC1/2 = histone deacetylase 1/2; MTA2 = metastasis‐associated protein 2; MECP2 = methyl‐CpG binding protein 2; LSD1 = lysine‐specific demethylase 1 (see Figure 1 for other definitions).

We then prioritized TFs for validation among those showing differential binding. We reasoned that as a regulatory complex of TFs, protein–protein interactions would be important. We therefore performed protein association network analysis (using the STRING database, version 11.0; www.strin​g‐db.org) and found several significant protein–protein interactions among the 38 factors identified (Figure 2A), with significant GO terms for regulation of IFN production (FDR 0.04) and NuRD complex (FDR 0.02). These analyses provided evidence of interactions involving IRF5, IKZF3 (the zinc‐finger protein Aiolos), RBBP4, and CHD4 (Figure 2A). Crucially, we were able to identify all of the previously described members of the NuRD repressor complex in our pull‐down experiments (Figure 2B and Supplementary Table 4, available on the *Arthritis & Rheumatology* website at http://onlin​e​libr​ary.wiley.com/doi/10.1002/art.41628/​abstract) (22).

To investigate this further, we analyzed DNA binding motifs spanning *rs4648889*. We found evidence of specific motifs for IKZF3 and CHD4 overlapping the disease‐associated A allele at *rs4648889* (Figure 2C). We interrogated publicly available ENCODE ChIP‐seq data (https://genome.ucsc.edu/ENCOD​E/) and found evidence of binding of both IKZF and CHD4 at or near these sites in lymphoblastoid cell lines (Figure 2D). Our label‐free qMS experiment showed that IKZF3 was significantly more abundant from pull‐down with the AS‐risk A allele than with the protective G allele (3.7‐fold increase; *P* < 0.03) (Figure 2E and Supplementary Table 4 [http://onlin​elibr​ary.wiley.com/doi/10.1002/art.41628/​abstract]). Binding to the A allele was also significantly increased for several components of the NuRD complex, including CHD4 (3.6‐fold increase; *P* < 0.05), RBBP4 (4.1‐fold increase; *P* < 0.03), and methyl‐CpG binding protein 2 (MECP2) (1.5‐fold increase; *P* = 0.05). Although not statistically significant, this trend of increased binding to the A allele continued for other NuRD proteins, including lysine‐specific histone demethylase 1 (KDM1A) and the histone deacetylases HDAC1 and HDAC2 (increase in preferential binding to the A allele of 2.4‐fold, 16.1‐fold, and 16.2‐fold, respectively) (Figure 2E and Supplementary Table 4).

#### Validation of differential binding for IKZF3, CHD4, and RBBP4

**Figure 3:**
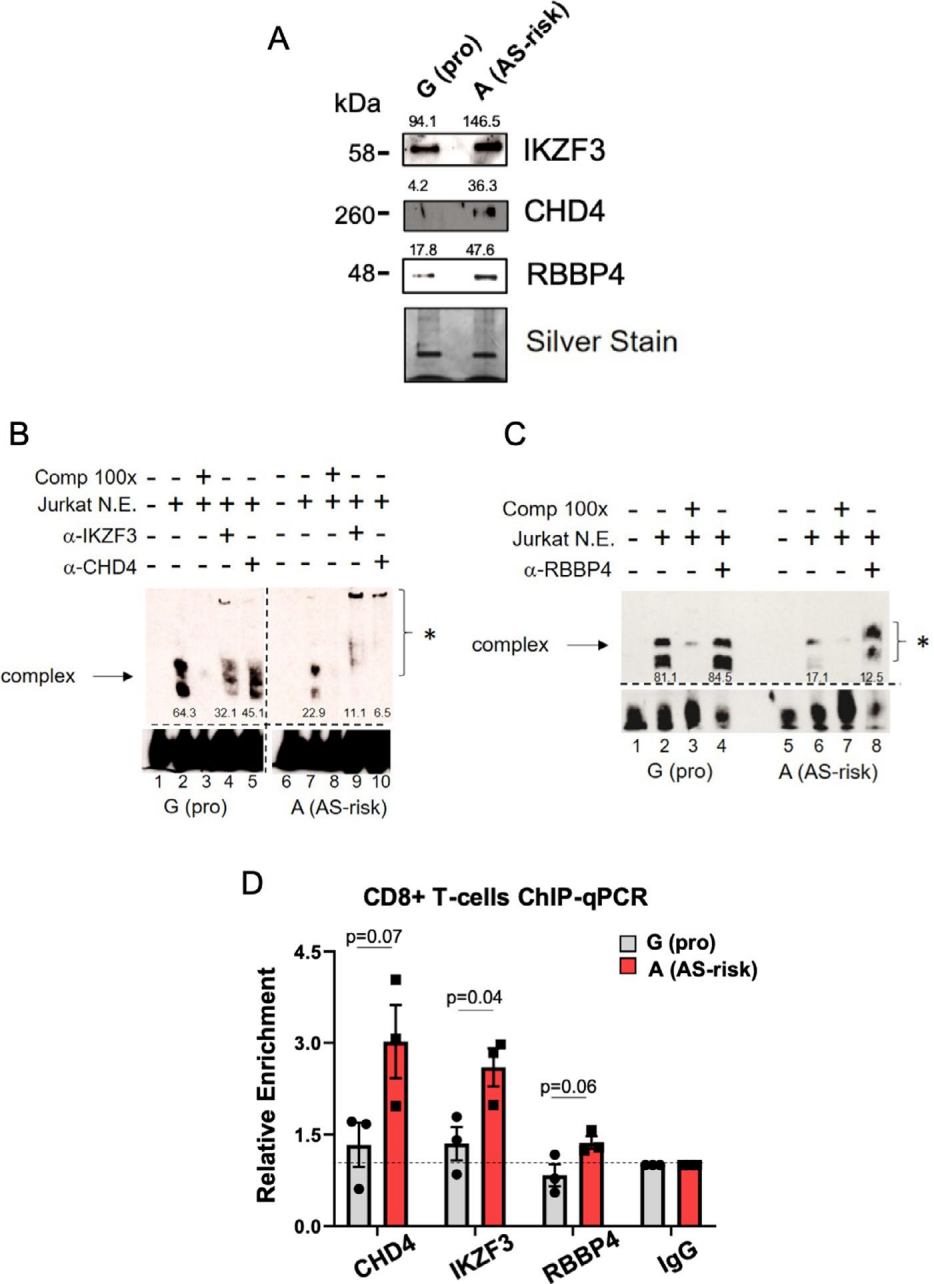
Validation of the NuRD factors identified. **A**, Representative Western blot (3 samples analyzed) of the pulled‐down eluates used in qMS experiments shows the differential binding of IKZF3, CHD4, and RBBP4 to the AS‐protective (pro) G allele versus the AS‐risk A allele. Silver staining was used for equal loading. Numbers above the bands show the quantification of binding, measured with ImageJ. **B**, Representative findings from electrophoretic mobility shift assay (EMSA) (2 samples analyzed) show differential nuclear extract (N.E.) binding after addition of Jurkat cell lysates (lanes 2 and 7). A 100‐fold excess of unlabeled probes was used as competitor (Comp) (lanes 3 and 8). The involvement of IKZF3 and CHD4 was assessed by adding the corresponding antibody (lanes 4, 5, 9, and 10). Numbers below the bands represent the pixel intensity, measured with ImageJ. **C**, Representative findings from EMSA (2 samples analyzed) show differential nuclear extract binding of RBBP4 after addition of the RBBP4 antibody (lanes 4 and 8). In **B** and **C**, the **asterisk** indicates the presence of a supershifted complex (**arrow**). **D**, The relative enrichment of CHD4, IKZF3, and RBBP4 was assessed with chromatin immunoprecipitation–quantitative polymerase chain reaction (ChIP‐qPCR) (3 samples analyzed) on CD8+ T cells heterozygous for *rs4648889* (from buffy coat blood cones). Data were normalized against a 1% input control, with IgG set at 1.0. Symbols represent individual samples; bars show the mean ± SD. *P* values were determined by Student’s *t*‐test. See Figure 1 for other definitions.

We then sought to validate our qMS results further. First, we used Western blots to analyze the pulled‐down eluates from the qMS experiments. We demonstrated increased amounts of IKZF3, CHD4, and RBBP4 in eluates pulled down with probes containing the AS‐risk A allele (Figure 3A).

We then determined the impact of *rs4648889* on TF binding by analyzing nuclear extracts from Jurkat T cells using EMSA. While the overall binding intensity of the DNA–nuclear extract complex was less with the A allele than with the G allele (units of intensity, mean ± SD 21.3 ± 3.7 with the A allele versus 68.1 ± 1.5 with the G allele [n = 3]; *P* = 0.002), incubation with antibodies against IKZF3, CHD4, and RBBP4 resulted in supershifted bands, which was more evident with the A allele (Figures 3B and C; see also Supplementary Figure 1A, available on the *Arthritis & Rheumatology* website at http://onlin​elibr​ary.wiley.com/doi/10.1002/art.41628/​abstract). These findings confirm the interaction of these TFs with the 50‐bp sequence encompassing *rs4648889*, and demonstrate increased binding of these TFs to the AS‐risk A allele.

We also performed allele‐specific ChIP‐qPCR to assess the relative abundance of these 3 specific factors. Freshly isolated CD8+ T cells from healthy donors who were heterozygous for *rs4648889* showed enhanced relative enrichment of binding to the AS‐risk A allele for CHD4, IKZF3, and RBBP4 (in 3 independent experiments) (*P* = 0.04, *P* = 0.07, and *P* = 0.06, respectively) (Figure 3D).

#### Preferential binding of IRF5 to the G allele at *rs4648889*

**Figure 4:**
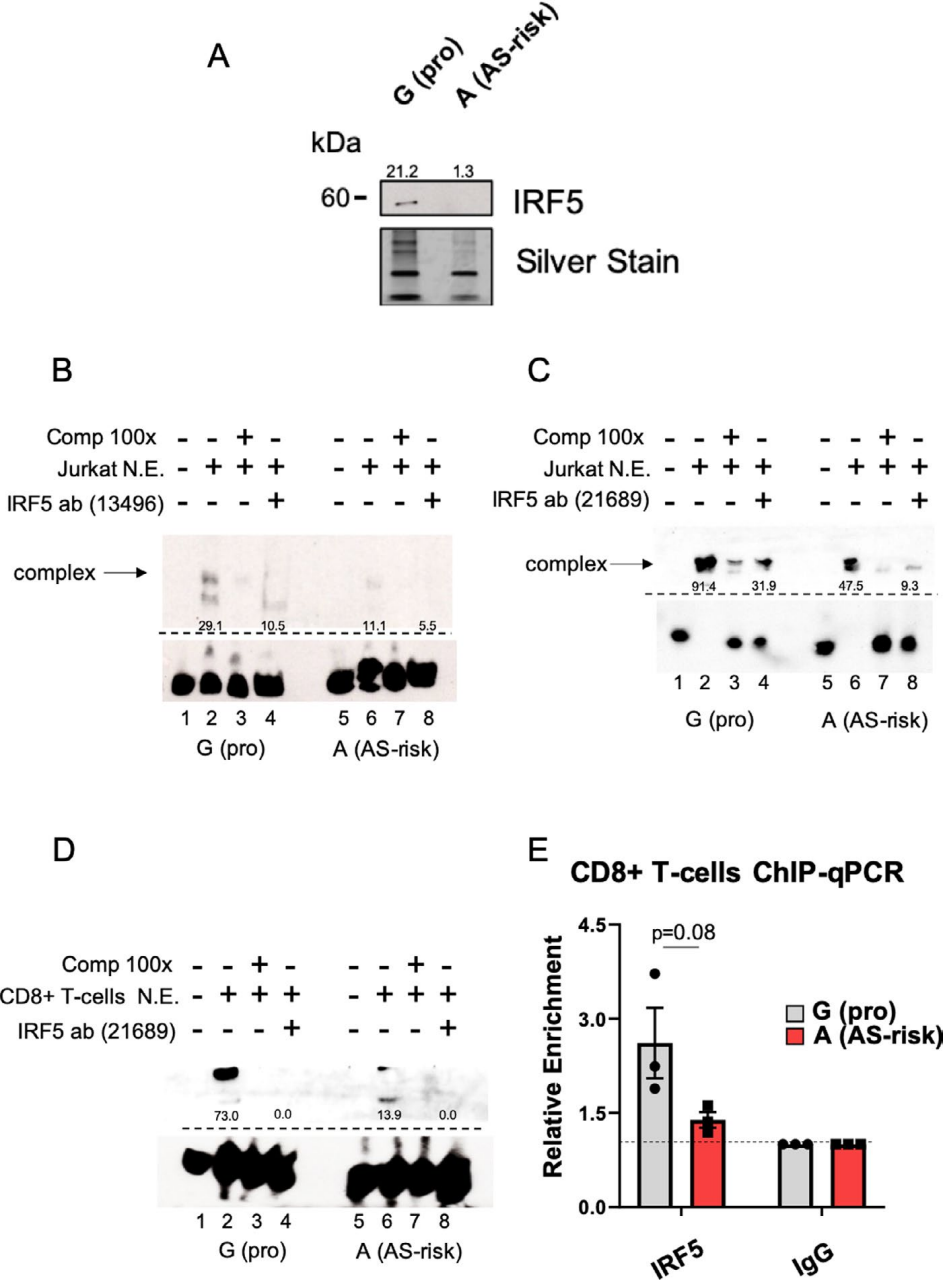
Validation of IRF5. **A**, Representative Western blot (3 samples analyzed) of the pulled‐down eluates used in qMS experiments shows the differential binding of IRF5 to the AS‐protective (pro) G allele versus AS‐risk A allele. Silver staining was used for equal loading. Numbers above the bands show the quantification of binding, measured with ImageJ. **B**, Representative findings from electrophoretic mobility shift assay (EMSA) (2 samples analyzed) show differential Jurkat cell nuclear extract (N.E.) binding for IRF5, after the addition of IRF5 antibody 13496 (lanes 4 and 8). In this case, inhibition of the complex was detected. **C** and **D**, Representative findings from EMSA (3 samples analyzed) show differential Jurkat cell (**C**) and CD8+ T cell (**D**) nuclear extract binding for IRF5, after addition of IRF5 antibody 21689 (lanes 4 and 8). Inhibition of the complex (**arrow**) is confirmed. **E**, The relative enrichment of IRF5 was assessed with allele‐specific chromatin immunoprecipitation–quantitative polymerase chain reaction (ChIP‐qPCR) on CD8+ T cells heterozygous for *rs4648889* (3 samples analyzed). Data were normalized against a 1% input control, with IgG set at 1.0. Symbols represent individual samples; bars show the mean ± SD. *P* values were determined by Student’s *t*‐test. See Figure 1 for other definitions.

Similar experimental approaches were used to validate IRF5 binding. Expression of IRF5 was evaluated by Western blotting in Jurkat T cells and CD8+ T cells (see Supplementary Figure 2, available on the *Arthritis & Rheumatology* website at http://onlin​elibr​ary.wiley.com/doi/10.1002/art.41628/​abstract). There was a markedly increased amount of IRF5 in eluates pulled down with probes containing the AS‐protective G allele compared to those containing the AS‐risk A allele (Figure 4A). Among the 38 proteins displaying significant differential allelic binding by qMS, IRF5 was significantly enriched in the fraction pulled down with the oligonucleotide containing the AS‐protective G allele (fold change 8.2; *P* = 0.003).

Similar to the above findings, EMSA revealed markedly greater binding of nuclear extracts from Jurkat T cells to the G allele, which was significantly reduced by preincubating the nuclear extracts with 2 independently validated anti‐IRF5 antibodies (Figures 4B and C; quantification of the Western blot findings is shown in Supplementary Figure 1B [http://onlin​elibr​ary.wiley.com/doi/10.1002/art.41628/​abstract]), although no supershift was seen in this case. Furthermore, EMSA analysis of nuclear extracts from CD8+ T cells confirmed this result, as shown in Figure 4D. These findings are consistent with the notion that IRF5 is involved in the DNA–protein complex. ChIP‐qPCR analysis of freshly isolated CD8+ T cells from healthy donors heterozygous for *rs4648889* also showed a trend toward enhanced enrichment of IRF5 for the protective G allele (*P* = 0.08) (Figure 4E). These findings suggest that an allelic imbalance occurs in these cells.

#### Impact of *IRF5* silencing on CD8+ T cells

**Figure 5:**
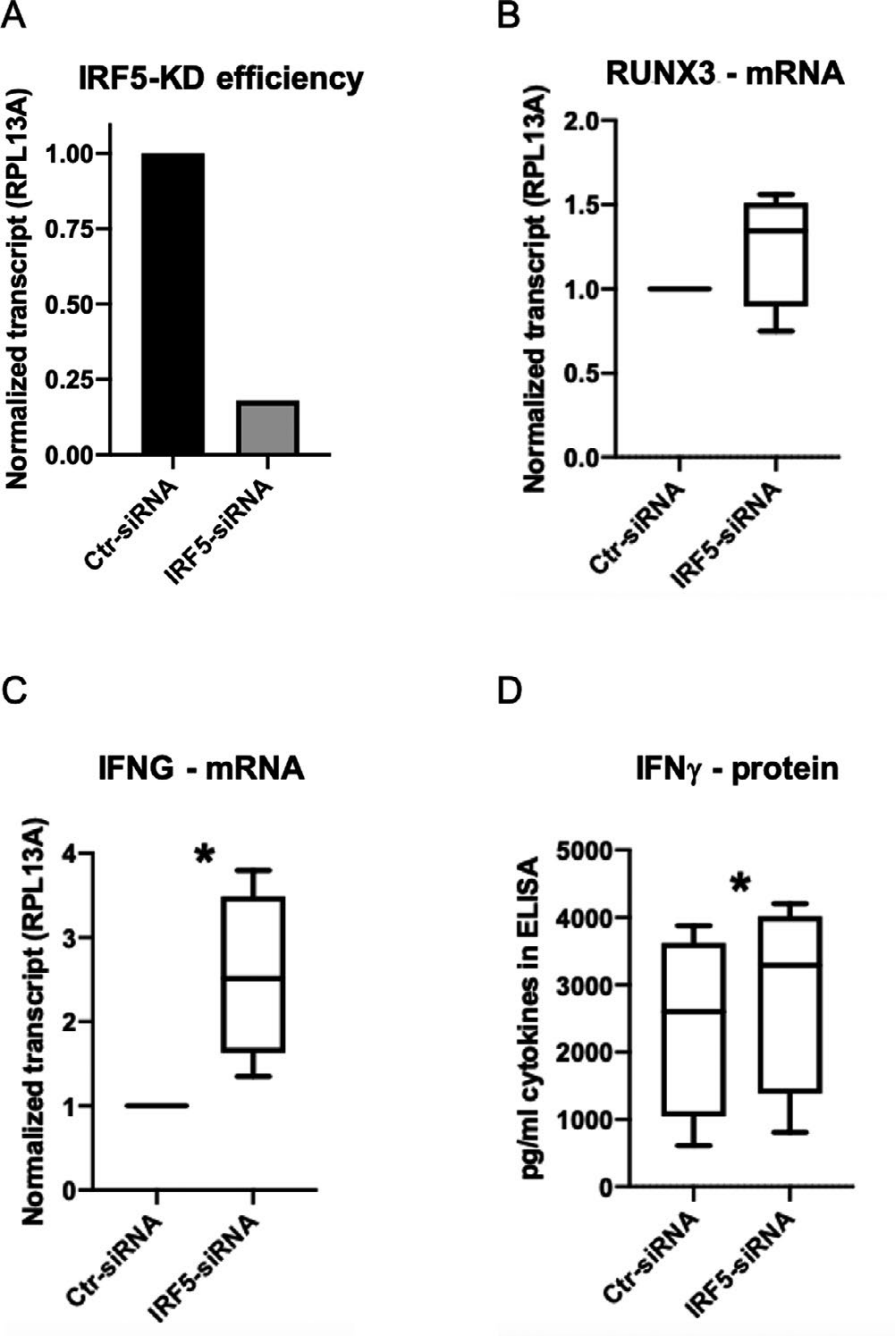
Functional validation of the role of interferon regulatory factor 5 (IRF5) using gene silencing. **A**, The efficiency of *IRF5* silencing was evaluated with reverse transcription–quantitative polymerase chain reaction (RT‐qPCR) on CD8+ T cells transfected with *IRF5* small interfering RNA (siRNA). A scrambled siRNA was used as control (Ctr‐siRNA). Results are shown as the normalized transcript levels after *IRF5* knockdown (KD) relative to control. **B**, Results of RT‐qPCR show the effect on *RUNX3* mRNA expression in CD8+ T cells after *IRF5* silencing (n = 4). **C**, Results of RT‐qPCR show the effect on expression of the interferon‐γ (IFNγ) gene after *IRF5* silencing in CD8+ T cells (n = 4). **D**, Results of enzyme‐linked immunosorbent assay (ELISA) show the effect on IFNγ protein levels in CD8+ T cells after *IRF5* knockdown (n = 4). Values in **B**–**D** are shown as box plots, where lines inside the box represent the median, the boxes show the interquartile range, and the lines outside the boxes show the 10th and 90th percentiles. * = *P* = 0.002 by Student’s *t*‐test.

We then investigated the potential functional significance of differential IRF5 binding in CD8+ T cells. We used siRNA to knock down *IRF5* expression in primary CD8+ T cells activated with anti‐CD2/anti‐CD3/anti‐CD28 beads. Transfection of CD8+ T cells with siRNA targeting *IRF5* resulted in a significant reduction (up to 91%) in *IRF5* expression compared to the effects of a scrambled control siRNA (Figure 5A). We initially observed a small, but nonsignificant, increase in *RUNX3* expression following IRF5 knockdown (Figure 5B). In addition, we observed a significant increase in the levels of both *IFNG* mRNA (*P* = 0.03) and IFNγ protein (*P* = 0.02) following *IRF5* knockdown in CD8+ T cells (Figures 5C and D).

## DISCUSSION

Converting knowledge obtained from GWAS for complex traits into a mechanistic understanding of the underlying pathologic processes represents a truly formidable challenge (23). In the present study we have shown the power of qMS to address this challenge, by using this approach to identify a complex network of TFs and chromatin regulatory proteins (24, 25) interacting with a putative *cis*‐regulatory (enhancer) element upstream of the distal *RUNX3* promoter, which has previously been implicated in the etiology of AS when investigated in GWAS and fine‐mapping studies (2, 5, 8). We find that many of these TFs exhibit differential allelic binding in vitro to a short DNA sequence flanking *rs4648889*. In particular, one member of the Ikaros family of closely related TFs known as Aiolos (IZKF3), a global regulator of chromatin architecture (26), binds preferentially to the AS‐risk A allele. The Ikaros family of TFs (including Aiolos) plays important roles in lymphocyte biology, and Aiolos has been incriminated in B cell disorders (hyperproliferative states, autoantibody production, and lymphomas), T cell proliferation, Th17 cell differentiation, and innate immune cell plasticity (27, 28, 29).

Aiolos is often physically associated with the NuRD complex, an ATP‐dependent chromatin‐remodeling complex involved in transcriptional repression (30, 31). We identified most of the components of the NuRD complex active at this locus (CHD4, MECP2, RBBP4, HDAC1, HDAC2, and KDM1A), all of which exhibit some degree of preferential binding to the A allele. These findings were consistent with our results from Western blotting, EMSA, and ChIP‐qPCR and suggest that the NuRD complex is recruited more efficiently to the AS‐associated A allele of *rs4648889* in the regulatory sequence upstream of *RUNX3*. This could account for our previous findings of the transcriptional repression of RUNX3 by the A allele of rs4648889 (8). CHD4 and the NuRD complex are pivotal in early T cell development, specifically during the transition from double‐negative to double‐positive (CD4+CD8+) T cell precursors (32, 33, 34). Moreover, CHD4 and the NuRD complex work together with IKZF family members in modifying *CD8a* transcription and expression (35). Of passing interest to rheumatologists, CHD4 is one of the target antigens for anti‐Mi2 autoantibodies, which are found in ~20% of patients with dermatomyositis (36). However, the relevance of these antibodies to the pathogenesis of myositis or the malignancies commonly associated with dermatomyositis is unclear.

The presence of IRF5 among the 38 factors exhibiting significant differential allelic binding to this region is intriguing, particularly since its binding was significantly higher to the protective G allele—in contrast to Aiolos and the NuRD complex, which bound preferentially to the A allele (as described above). Our hypothesis‐free DNA pull‐down approach clearly demonstrated the differential binding of IRF5 to the risk and protective alleles of *rs4648889*. The detection of a unique peptide (LITVQVVPVAAR) in all 3 replicated pull‐down experiments with the protective allele demonstrates categorically the presence of IRF5, as has also recently been shown by others (37). These results were also independently supported by the observed changes in IRF5 expression levels evaluated on the pull‐down eluates from Western blotting and as analyzed with in vitro supershift assays performed with Jurkat and CD8+ T cells.

We corroborated our findings by using 2 different IRF5‐specific antibodies. IRF5 is one of the closely related members of the IRF family that play critical roles in cell differentiation, development, and proliferation. It is constitutively expressed in monocytes, macrophages, B cells, and dendritic cells (38) and is a key factor in promoting polarization toward the inflammatory (M1) macrophage phenotype, which subsequently enhances the development of Th1–Th17 cell responses (39). It plays a central role in the induction of inflammatory cytokines (40) and in determining macrophage responses to stimulation by IFNγ and granulocyte–macrophage colony‐stimulating factor, which appears to be a key factor in the pathogenesis of AS (41). Stimulation by Toll‐like receptor and Fcγ receptor are both required for IRF5 phosphorylation, activation, and nuclear translocation, which are essential for its transcription (42). The role of IRF5 in T cells has been explored relatively rarely, in contrast to its well‐defined actions in monocytes/macrophages. Recently, a possible role for IRF5 in the differentiation and migration of CD4+ and CD8+ T cells, and their production of cytokines, has been described (43). IRF5 is up‐regulated in mouse splenic T cells during chronic infection with *Leishmania donovani* (44) and has also been strongly associated genetically with several immune‐mediated diseases (9, 45).

The increased production of IFNγ by CD8+ lymphocytes that we observed after *IRF5* silencing was particularly interesting, in view of another recent study relating to chronic visceral leishmaniasis in mice. In these animals, IFNγ‐producing CD4+ T cells were more abundant in the spleen of an *irf5^−/−^* mouse, in which increased IRF5 expression was triggered through Toll‐like receptor 7, and CD4+ T cells were sensitized to cell death by increased expression of death receptor 5, indicating a possible mechanism for the maintenance of chronic infection (46). However, it is not known whether IRF5 might have a similar role in CD8+ T cells. Since the enhancer region that was studied herein lies closest to *RUNX3*, we assumed that it is most likely to influence expression of this gene. However, although we demonstrated a small increase in *RUNX3* expression following *IRF5* knockdown, this was not conclusive and a statistically significant change was not observed. It is possible that any regulatory effects involving IRF5 at this locus might also involve other TFs; this will need further evaluation. However, in mice, *irf5* has been showed to have a regulatory effect on *Runx3* transcription in CD11+ intestinal macrophages, although data from T cells are so far lacking (47).

The therapeutic possibilities with regard to modulation of IRF5 expression, its posttranslational modification, and/or functional interactions with its protein partners have been extensively discussed elsewhere (39, 48). For example, AAAG‐rich oligodeoxynucleotides that compete with IRF5 for the consensus DNA binding site in regulatory elements associated with inflammatory genes have been used successfully to ameliorate bacterial septic peritonitis and influenza‐induced acute lung injury and to reduce the levels of IL‐6, tumor necrosis factor, and type 2 nitric oxide synthase in mouse models (49, 50).

The experiments described herein represent a continuation of our approach to discover the pathogenic mechanisms underlying the strong genetic association of AS with RUNX3. In this study and in our previous work (8), we have demonstrated the impact of AS‐associated SNPs on TF binding to the enhancer‐like region upstream of *RUNX3* and on RUNX3 expression in CD8+ T cells (8). Further clarification of the involved pathways is needed, but these results suggest that *RUNX3*‐ and IRF5‐related pathways represent important potential therapeutic targets for the treatment of AS and support the role of CD8 lymphocytes in its pathology.

## AUTHOR CONTRIBUTIONS

All authors were involved in drafting the article or revising it critically for important intellectual content, and all authors approved the final version to be published. Dr. Vecellio had full access to all of the data in the study and takes responsibility for the integrity of the data and the accuracy of the data analysis.

### Study conception and design

Vecellio, Chen, Cohen, Cortes, Fischer, Knight, Wordsworth.

### Acquisition of data

Li, Bonham, Fischer.

### Analysis and interpretation of data

Vecellio, Chen, Selmi, Brown, Fischer.

## Supporting information

Fig S1‐S2

Table S1‐S4

## References

[1] Cortes A , Pulit SL , Leo PJ , Pointon JJ , Robinson PC , Weisman MH , et al. Major histocompatibility complex associations of ankylosing spondylitis are complex and involve further epistasis with ERAP1. Nat Commun 2015;6:7146. 25994336 10.1038/ncomms8146 PMC4443427

[2] Cortes A , Hadler J , Pointon JJ , Robinson PC , Karaderi T , Leo P , et al, on behalf of the International Genetics of Ankylosing Spondylitis Consortium . Identification of multiple risk variants for ankylosing spondylitis through high‐density genotyping of immune‐related loci. Nat Genet 2013;45:730–40. 23749187 10.1038/ng.2667 PMC3757343

[3] Ellinghaus D , Jostins L , Spain SL , Cortes A , Bethune J , Han B , et al. Analysis of five chronic inflammatory diseases identifies 27 new associations and highlights disease‐specific patterns at shared loci. Nat Genet 2016;48:510–8. 26974007 10.1038/ng.3528 PMC4848113

[4] Di Meglio P , Villanova F , Napolitano L , Tosi L , Barberio MT , Mak RK , et al. The IL23R A/Gln381 allele promotes IL‐23 unresponsiveness in human memory T‐helper 17 cells and impairs Th17 responses in psoriasis patients. J Invest Dermatol 2013;133:2381–9. 23563201 10.1038/jid.2013.170 PMC3778837

[5] Evans DM , Spencer CC , Pointon JJ , Su Z , Harvey D , Kochan G , et al. Interaction between ERAP1 and HLA–B27 in ankylosing spondylitis implicates peptide handling in the mechanism for HLA–B27 in disease susceptibility. Nat Genet 2011;43:761–7. 21743469 10.1038/ng.873 PMC3640413

[6] Kochan G , Krojer T , Harvey D , Fischer R , Chen L , Vollmar M , et al. Crystal structures of the endoplasmic reticulum aminopeptidase‐1 (ERAP1) reveal the molecular basis for N‐terminal peptide trimming. Proc Natl Acad Sci U S A 2011;108:7745–50. 21508329 10.1073/pnas.1101262108 PMC3093473

[7] Hindorff LA , Sethupathy P , Junkins HA , Ramos EM , Mehta JP , Collins FS , et al. Potential etiologic and functional implications of genome‐wide association loci for human diseases and traits. Proc Natl Acad Sci U S A 2009;106:9362–7. 19474294 10.1073/pnas.0903103106 PMC2687147

[8] Vecellio M , Roberts AR , Cohen CJ , Cortes A , Knight JC , Bowness P , et al. The genetic association of RUNX3 with ankylosing spondylitis can be explained by allele‐specific effects on IRF4 recruitment that alter gene expression. Ann Rheum Dis 2016;75:1534–40. 26452539 10.1136/annrheumdis-2015-207490 PMC4975853

[9] Duarte JH . IRF5 mediates joint inflammation [review]. Nat Rev Rheumatol 2015;11:562. 10.1038/nrrheum.2015.127 26369611

[10] Eames HL , Corbin AL , Udalova IA . Interferon regulatory factor 5 in human autoimmunity and murine models of autoimmune disease [review]. Transl Res 2016;167:167–82. 26207886 10.1016/j.trsl.2015.06.018

[11] Weiss M , Blazek K , Byrne AJ , Perocheau DP , Udalova I . IRF5 is a specific marker of inflammatory macrophages in vivo. Mediators Inflamm 2013;2013:245804. 24453413 10.1155/2013/245804 PMC3885211

[12] Levanon D , Negreanu V , Lotem J , Bone KR , Brenner O , Leshkowitz D , et al. Transcription factor Runx3 regulates interleukin‐15‐dependent natural killer cell activation. Mol Cell Biol 2014;34:1158–69. 24421391 10.1128/MCB.01202-13 PMC3958033

[13] Lotem J , Levanon D , Negreanu V , Bauer O , Hantisteanu S , Dicken J , et al. Runx3 at the interface of inflammation, immunity and cancer [review]. Biochim Biophys Acta 2015;1855:131–43. 25641675 10.1016/j.bbcan.2015.01.004

[14] Tacheny A , Michel S , Dieu M , Payen L , Arnould T , Renard P . Unbiased proteomic analysis of proteins interacting with the HIV‐1 5′LTR sequence: role of the transcription factor Meis. Nucleic Acids Res 2012;40:e168. 22904091 10.1093/nar/gks733 PMC3505963

[15] Caballero R , Setien F , Lopez‐Serra L , Boix‐Chornet M , Fraga MF , Ropero S , et al. Combinatorial effects of splice variants modulate function of Aiolos. J Cell Sci 2007;120:2619–30. 17646674 10.1242/jcs.007344

[16] Zhang J , Jackson AF , Naito T , Dose M , Seavitt J , Liu F , et al. Harnessing of the nucleosome‐remodeling‐deacetylase complex controls lymphocyte development and prevents leukemogenesis. Nat Immunol 2011;13:86–94. 22080921 10.1038/ni.2150 PMC3868219

[17] Sepil I , Hopkins BR , Dean R , Thézénas ML , Charles PD , Konietzny R , et al. Quantitative proteomics identification of seminal fluid proteins in male drosophila melanogaster. Mol Cell Proteomics 2019;18:S46–58. 30287546 10.1074/mcp.RA118.000831 PMC6427238

[18] Fischer R , Kessler BM . Gel‐aided sample preparation (GASP): a simplified method for gel‐assisted proteomic sample generation from protein extracts and intact cells. Proteomics 2015;15:1224–9. 25515006 10.1002/pmic.201400436 PMC4409837

[19] Jones P , Côté RG , Martens L , Quinn AF , Taylor CF , Derache W , et al. PRIDE: a public repository of protein and peptide identifications for the proteomics community. Nucleic Acids Res 2006;34:D659–63. 16381953 10.1093/nar/gkj138 PMC1347500

[20] Fabregat A , Jupe S , Matthews L , Sidiropoulos K , Gillespie M , Garapati P , et al. The Reactome Pathway Knowledgebase. Nucleic Acids Res 2018;46:D649–55. 29145629 10.1093/nar/gkx1132 PMC5753187

[21] Bernstein BE , Stamatoyannopoulos JA , Costello JF , Ren B , Milosavljevic A , Meissner A , et al. The NIH Roadmap Epigenomics Mapping Consortium. Nat Biotech 2010;28:1045–8. 10.1038/nbt1010-1045 PMC3607281 20944595

[22] Millard CJ , Varma N , Saleh A , Morris K , Watson PJ , Bottrill AR , et al. The structure of the core NuRD repression complex provides insights into its interaction with chromatin. Elife 2016;5:e13941. 27098840 10.7554/eLife.13941 PMC4841774

[23] Claussnitzer M , Cho JH , Collins R , Cox NJ , Dermitzakis ET , Hurles M , et al. A brief history of human disease genetics [review]. Nature 2020;577:179–89. 31915397 10.1038/s41586-019-1879-7 PMC7405896

[24] Hu S , Xie Z , Onishi A , Yu X , Jiang L , Roo H , et al. Profiling the human protein‐DNA interactome reveals ERK2 as a transcriptional repressor of interferon signalling. Cell 2009;139:610–22. 19879846 10.1016/j.cell.2009.08.037 PMC2774939

[25] Vaquerizas JM , Kummerfeld SK , Teichmann SA , Luscombe NM . A census of human transcription factors: function, expression and evolution [review]. Nat Rev Genet 2009;10:252–63. 19274049 10.1038/nrg2538

[26] Kim J , Sif S , Jones B , Koipally J , Heller E , Winandy S , et al. Ikaros DNA‐binding proteins direct formation of chromatin remodeling complexes in lymphocytes. Immunity 1999;10:345–55. 10204490 10.1016/s1074-7613(00)80034-5

[27] Quintana FJ , Jin H , Burns EJ , Nadeau M , Yeste A , Kumar D , et al. Aiolos promotes Th17 differentiation by directly silencing Il2 expression. Nat Immunol 2012;13:770–7. 22751139 10.1038/ni.2363 PMC3541018

[28] Heizmann B , Kastner P , Chan S . The Ikaros family in lymphocyte development. Curr Op Immunol 2018;51:14–23. 10.1016/j.coi.2017.11.005 29278858

[29] Wang JH , Avitahl N , Cariappa A , Friedrich C , Ikeada T , Renola A , et al. Aiolos regulates B cell activation and maturation to effector state. Immunity 1998;9:543–53. 9806640 10.1016/s1074-7613(00)80637-8

[30] Koipally J , Renold A , Kim J , Georgopoulos K . Repression by Ikaros and Aiolos is mediated through histone deacetylase complexes. EMBO J 1999;18:3090–100. 10357820 10.1093/emboj/18.11.3090 PMC1171390

[31] Bornelöv S , Reynolds N , Xenophontos M , Gharbi S , Johnstone E , Floyd R , et al. The nucleosome remodeling and deacetylation complex modulates chromatin structure at sites of active transcription to fine‐tune gene expression. Mol Cell 2018;71:56–72. 30008319 10.1016/j.molcel.2018.06.003 PMC6039721

[32] Istaces N , Splittberger M , Silva VL , Nguyen M , Thomas S , Le A , et al. EOMES interacts with RUNX3 and BRG1 to promote innate memory cell formation through epigenetic reprogramming. Nat Commun 2019;10:3306. 31341159 10.1038/s41467-019-11233-6 PMC6656725

[33] Dege C , Hagman J . Mi‐2/NuRD chromatin remodelling complexes regulate B and T‐lymphocyte development [review]. Immunol Rev 2014;261:126–40. 25123281 10.1111/imr.12209 PMC4386592

[34] Williams CJ , Naito T , Arco PG , Seavitt JR , Cashman SM , De Souza B , et al. The chromatin remodeler Mi‐2β is required for CD4 expression and T cell development. Immunity 2004;20:719–33. 15189737 10.1016/j.immuni.2004.05.005

[35] Harker N , Garefalaki A , Menzel U , Ktistaki E , Naito T , Georgopoulos K , et al. Pre‐TCR signalling and CD8 gene bivalent chromatin resolution during thymocyte development. J Immunol 2011;186:6368–77. 21515796 10.4049/jimmunol.1003567

[36] Ceribelli A , Isailovic N , De Santis M , Generali E , Fredi M , Cavazzana I , et al. Myositis‐specific autoantibodies and their association with malignancy in Italian patients with polymyositis and dermatomyositis. Clin Rheumatol 2017;36:469–75. 27761751 10.1007/s10067-016-3453-0

[37] Idborg H , Zandian A , Ossipova E , Wigren E , Preger C , Mobarrez F , et al. Circulating levels of interferon regulatory factor‐5 associates with subgroups of systemic lupus erythematosus patients. Front Immunol 2019;10:1029. 31156624 10.3389/fimmu.2019.01029 PMC6533644

[38] Barnes BJ , Richards J , Mancl M , Hanash S , Beretta L , Pitha PM . Global and distinct targets of IRF‐5 and IRF‐7 during innate response to viral infection. J Biol Chem 2004;45:194–207. 10.1074/jbc.M400726200 15308637

[39] Krausgruber T , Blazek K , Smallie T , Alzabin S , Lockstone H , Sahgal N , et al. IRF5 promotes inflammatory macrophage polarization and TH1‐TH17 responses. Nat Immunol 2011;12:231–9. 21240265 10.1038/ni.1990

[40] Almuttaqi H , Udalova I . Advances and challenges in targeting IRF5, a key regulator of inflammation [review]. FEBS J 2019;286:1624–37. 30199605 10.1111/febs.14654 PMC6563445

[41] Al‐Mossawi MH , Chen L , Fang H , Ridley A , de Wit J , Yager N , et al. Unique transcriptome signatures and GM‐CSF expression in lymphocytes from patients with spondyloarthritis. Nat Commun 2017;8:1510. 29142230 10.1038/s41467-017-01771-2 PMC5688161

[42] Hoepel W , Newling M , Vogelpoel LT , Sritharan L , Hansen IS , Kapsenberg ML , et al. FCyr‐TLR cross talk enhances THNF production by human monocyte‐derived DCs via IRF5‐dependent gene transcription and glycolytic reprogramming. Front Immunol 2019;10:739. 31024565 10.3389/fimmu.2019.00739 PMC6464031

[43] Yan J , Pandey SP , Barnes BJ , Turner JR , Abraham C . T cell‐intrinsic IRF5 regulates T cell signaling, migration, and differentiation and promotes intestinal inflammation. Cell Rep 2020;31:107820. 32610123 10.1016/j.celrep.2020.107820 PMC7409536

[44] Paun A , Bankoti R , Joshi T , Pitha PM , Stager S . Critical role of IRF‐5 in the development of T helper 1 responses to Leishmania donovani infection. PLoS Pathog 2011;7:e1001246. 21253574 10.1371/journal.ppat.1001246 PMC3017120

[45] Nordang GB , Viken MK , Amundsen SS , Sanchez ES , Flato B , Forre OT , et al. Interferon regulatory factor 5 gene polymorphism confers risk to several rheumatic diseases and correlates with expression of alternative thymic transcripts. Rheumatology (Oxford) 2012;51:619–26. 22179739 10.1093/rheumatology/ker364

[46] Fabie A , Mai LT , Dagenais‐Lussier X , Hammami A , van Grevenynghe J , Stager S . IRF‐5 promotes T cell death in CD4 cells during chronic infection. Cell Rep 2018;24:1163–75. 30067973 10.1016/j.celrep.2018.06.107

[47] Corbin AL , Gomez‐Vazquez M , Berthold DL , Attar M , Arnold IC , Powrie FM , et al. IRF5 guides monocytes toward an inflammatory CD11c+ macrophage phenotype and promotes intestinal inflammation. Sci Immunol 2020;47:eaax6085. 10.1126/sciimmunol.aax6085 PMC7611075 32444476

[48] Thompson C , Matta B , Barnes BJ . Therapeutic targeting of IRFs: pathway‐dependence or structure‐based? [review]. Frontiers Immunol 2018;9:2622. 10.3389/fimmu.2018.02622 PMC6255967 30515152

[49] Gao S , Li X , Nie S , Yang L , Tu L , Dong B , et al. An AAAG‐rich oligodeoxynucleotide rescues mice from bacterial septic peritonitis by interfering interferon regulatory factor 5. Int J Mol Sci 2017;18:1034. 10.3390/ijms18051034 PMC5454946 28492513

[50] Fang ML , Wan M , Guo S , Sun R , Yang M , Zhao T , et al. An oligodeoxynucleotide capable of lessening acute lung inflammatory injury in mice infected by influenza virus. Biochem Biophys Res Commun 2011;415:342–7. 22033400 10.1016/j.bbrc.2011.10.062

