# Supplementary material for: Functional Genomic Analysis of a RUNX3 Polymorphism Associated With Ankylosing Spondylitis
Source: Arthritis Rheumatol. 2021 May 2;73(6):980–90. doi: 10.1002/art.41628 (PMC8251554; doi:10.1002/art.41628)
Supplement: Supplementary file 1 — Fig S1‐S2 [file ART-73-980-s001.pdf]

Suppl. Figure 1

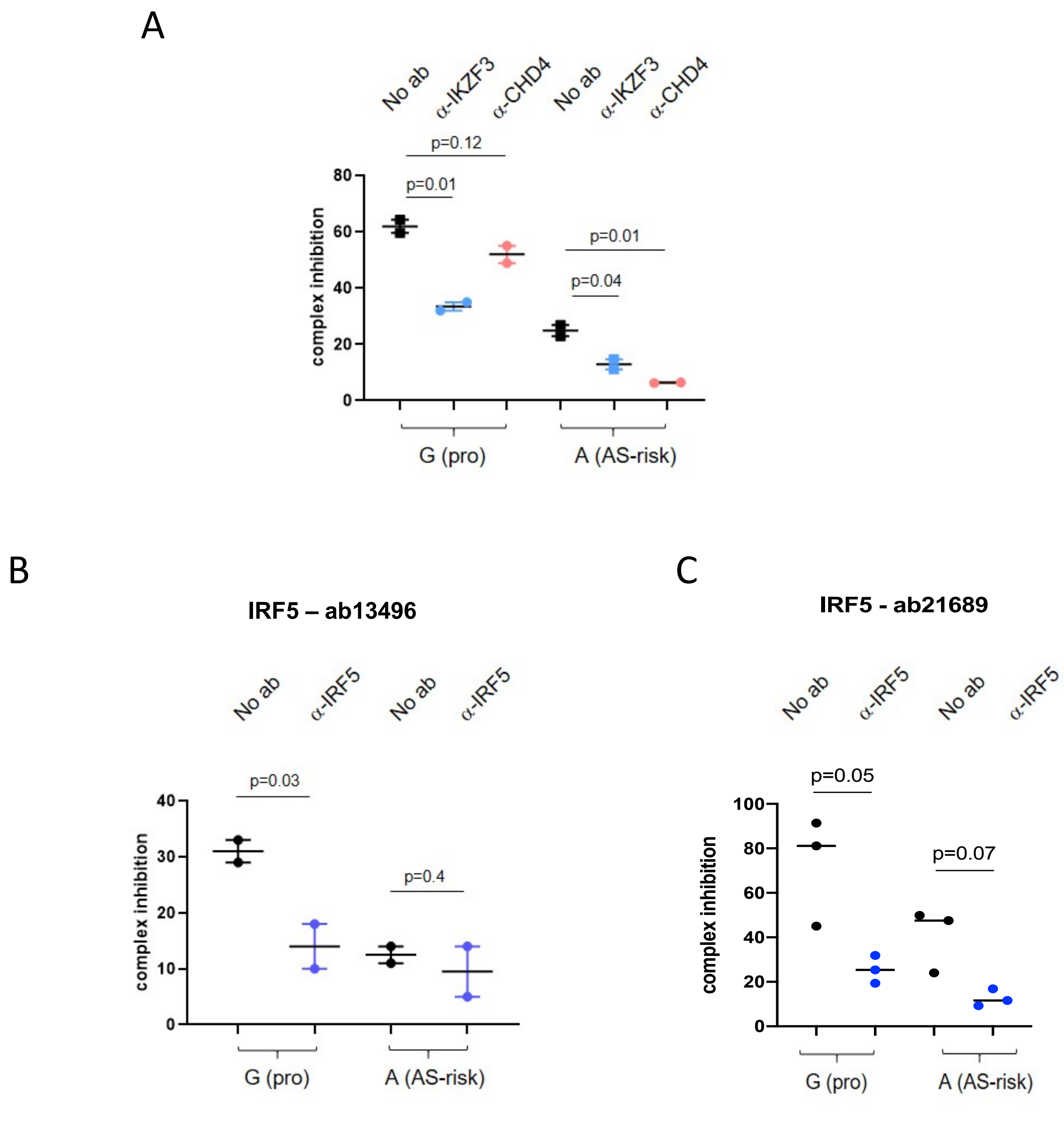

Supplementary Figure 1. (A) Quantification of the EMSA showing inhibition of complex formation after addition of IKZF3 and CHD4 antibodies (n=2) performed with Image J.
(B) Quantification of complex inhibition (with Jurkat NE) after addition of IRF5 antibody ab13496 (n=2) performed with Image J. (C) Quantification of complex inhibition (with Jurkat NE) after addition of IRF5 antibody ab21689 (n=3) performed with Image J.

Suppl. Figure 2

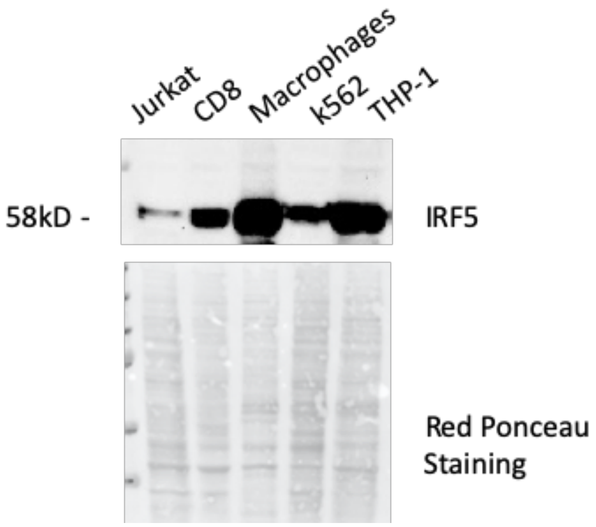

Supplementary Figure 2. Representative WB (n=3) showing IRF5 protein expression evaluated in Jurkat, CD8+ T-cells, macrophages, K562 erythroleukemia and THP-1 monocytic cells line, with antibody (anti-IRF5, ab21689). Red Ponceau staining shows same amount of protein loaded in each well.
