# Supplementary material for: Functional Genomic Analysis of a RUNX3 Polymorphism Associated With Ankylosing Spondylitis
Source: Arthritis Rheumatol. 2021 May 2;73(6):980–90. doi: 10.1002/art.41628 (PMC8251554; doi:10.1002/art.41628)
Supplement: Supplementary file 2 — Table S1‐S4 [file ART-73-980-s002.docx]

**Supplementary Table 1.** List of DNA probes for EMSA and pull-down experiments. Asterisks locate SNP *rs4648889*.

DNA probes:

| *rs4648889* Forward, A allele: 5’-CCT GAG GGG CTT CCC CCT CCC TGG A*AA CCT GAG  TCC AGG CCC AGG AAG G-3’,  *rs4648889* Reverse, A allele: 5’-CCT TCC TGG GGC CTG GAC TCA GGT TT*C CAG GGA  GGG GGA ACC CCT CAG G-3’;  *rs4648889* Forward, G allele: 5’- CCT GAG GGG CTT CCC CCT CCC TGG G*AA CCT  GAG TCC AGG CCC AGG AAG G-3’,  *rs4648889* Reverse, G allele: 5’- CCT TCC TGG GGC CTG GAC TCA GGT TC*C CAG  GGA GGG GGA ACC CCT CAG G -3’. |
| --- |

**Supplementary Table 2.** List of antibodies used for Western blot analysis.

Antibodies: Manufacturer: Dilution used:

| IKZF3 | Abcam (ab139408) | 1:20000 |
| --- | --- | --- |
| CHD4 | Proteintech Europe (14173-1-AP) | 1:1000 |
| RBBP4 | Abcam (ab79416) | 1:10000 |
| IRF5 | Cell Signaling (13496) | 1:1000 |
| IRF5 | Abcam (ab21689) | 1:1000 |
| Goat anti-mouse | Life technologies | 1:5000 |
| Goat anti-rabbit | Life technologies | 1:10000 |

**Supplementary Table 3. List of proteins with significantly differential binding affinity (p<0.05) to probes corresponding to the two alleles of *rs4648889***. Label-free quantification results for G (protective) and A (AS-risk) alleles, expressed as average, obtained from three independent experiments. Fold change and t-test are also shown. Proteins showing greater abundance with the G allele are highlighted in yellow while in light blue those for the A allele.


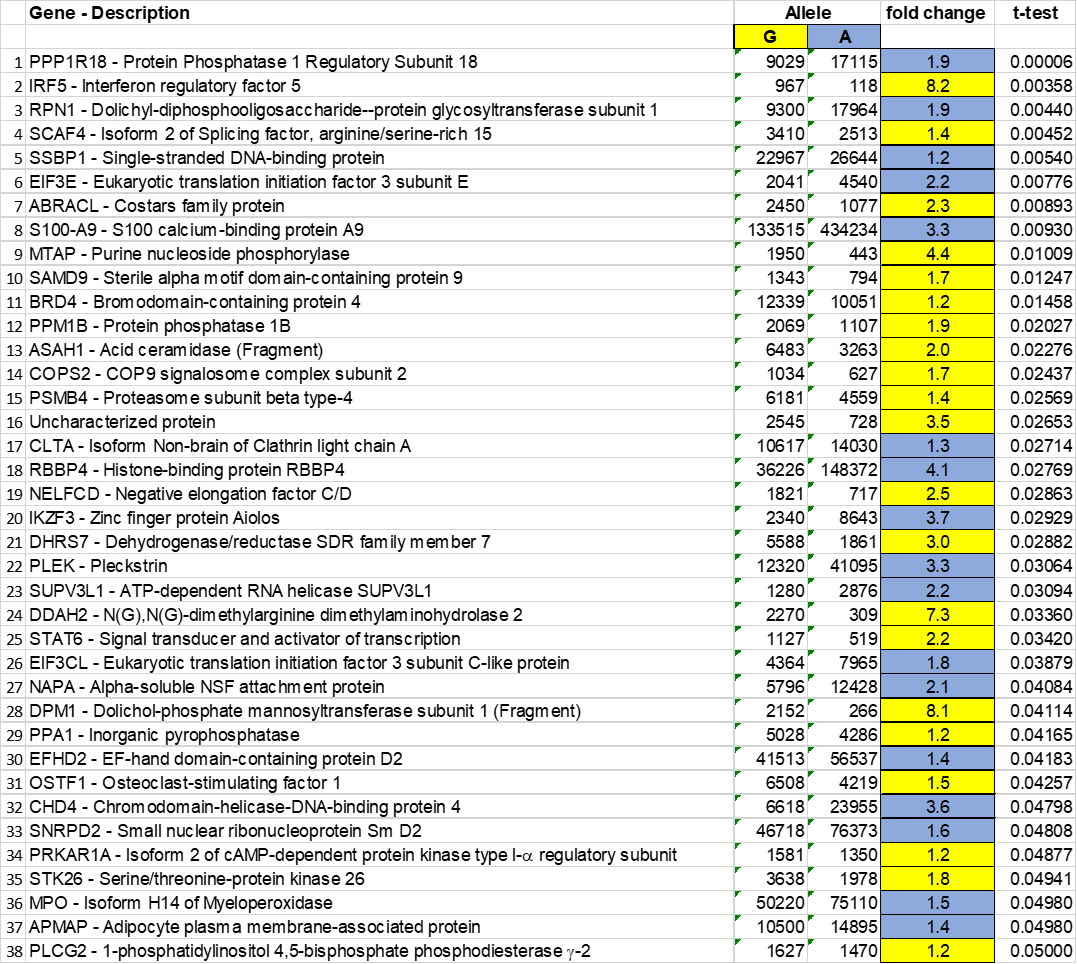


**Supplementary Table 4. Label-free quantification of IRF5, IKZF3 and NuRD complex factors identified with MS approach.** Label-free quantification after median subtraction of IKZF3, IRF5 and the NuRD complex factors obtained from three independent experiments. NuRD proteins showed greater abundance with the A allele, highlighted in light blue. IRF5 showed significantly more abundance with the G allele. The unique peptide number is also shown (as average of 3 experiments). *Significant p-value.

**
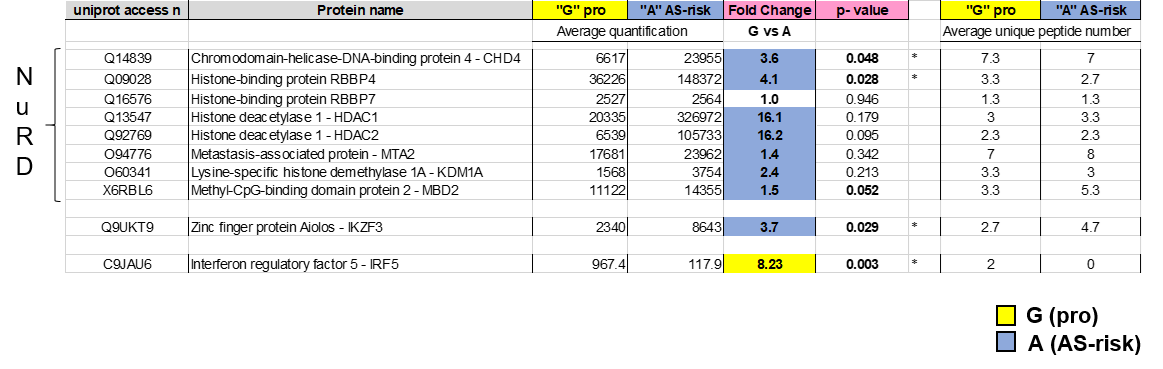
**
